# Effects of patterned electrical sensory nerve stimulation and static stretching on joint range of motion and passive torque

**DOI:** 10.3389/fnins.2023.1205602

**Published:** 2023-08-22

**Authors:** Akira Saito, Takamasa Mizuno

**Affiliations:** ^1^Center for Health and Science, Kyushu Sangyo University, Fukuoka, Japan; ^2^Research Center of Health, Physical Fitness and Sports, Nagoya University, Nagoya, Japan

**Keywords:** proprioceptive neuromuscular facilitation, reciprocal inhibition, ankle joint, Hoffmann-reflex, stretch tolerance, muscle stiffness

## Abstract

Static stretching and proprioceptive neuromuscular facilitation stretching techniques can modulate specific neural mechanisms to improve the range of motion. However, the effects of modulation of these neural pathways on changes in the range of motion with static stretching remain unclear. Patterned electrical stimulation of the sensory nerve induces plastic changes in reciprocal Ia inhibition. The present study examined the effects of patterned electrical stimulation and static stretching on a range of motion and passive torque in plantarflexion muscles. The subjects were 14 young men (age 20.8 ± 1.3 years). The effects of patterned electrical stimulation (10 pulses at 100 Hz every 1.5 s) or uniform electrical stimulation (one pulse every 150 ms) to the common peroneal nerve for 20 min on reciprocal Ia inhibition of the Hoffman reflex (H-reflex) were examined. Reciprocal Ia inhibition was evaluated as short-latency suppression of the soleus H-reflex by conditioning stimulation of the common peroneal nerve. Then, the effects of transcutaneous electrical nerve stimulation (patterned electrical stimulation or uniform electrical stimulation) or prolonged resting (without electrical stimulation) and static 3-min stretching on the maximal dorsiflexion angle and passive torque were investigated. The passive ankle dorsiflexion test was performed on an isokinetic dynamometer. Stretch tolerance and stiffness of the muscle-tendon unit were evaluated by the peak and slope of passive torques, respectively. Patterned electrical stimulation significantly increased reciprocal Ia inhibition of soleus H-reflex amplitude (9.7 ± 6.1%), but uniform electrical stimulation decreased it significantly (19.5 ± 8.8%). The maximal dorsiflexion angle was significantly changed by patterned electrical stimulation (4.0 ± 1.4°), uniform electrical stimulation (3.8 ± 2.3°), and stretching without electrical stimulation (2.1 ± 3.3°). The increase in stretch tolerance was significantly greater after patterned electrical stimulation and uniform electrical stimulation than after stretching without electrical stimulation. Stiffness of the muscle-tendon unit was significantly decreased by patterned electrical stimulation, uniform electrical stimulation, and stretching without electrical stimulation. Transcutaneous electrical nerve stimulation and static stretching improve stretch tolerance regardless of the degree of reciprocal Ia inhibition.

## 1. Introduction

Static stretching is widely used by athletes and in clinical settings to improve the flexibility of human joints. Maximal joint range of motion (ROM) and resistance to stretch (index of stiffness and stretch tolerance) are functional parameters that may affect muscle strain injury risk (Witvrouw et al., 2003) and are compromised with aging (Bassey et al., 1989). It has been suggested that improvement of ROM after static stretching can be attributed to several mechanisms, including reduction in stiffness of the muscle-tendon units (Blazevich et al., 2012; Mizuno et al., 2013), increased stretch tolerance (Magnusson et al., 1996b; Kay et al., 2016), and altered sensitivity of the stretch reflex (Avela et al., 1999; Guissard and Duchateau, 2006). As the underlying technical factors of static stretching that influence ROM and resistance to stretch, stretching intensity determines the effectiveness of static stretching (Freitas et al., 2015). Static stretching performed to increase ROM had a greater effect on the maximal tolerable joint angle than on the submaximal angle (Kataura et al., 2017). Therefore, static stretching should be performed at the maximal tolerable intensity to obtain greater benefits for joint flexibility.

It has been reported that proprioceptive neuromuscular facilitation (PNF) stretching is more effective for increasing ROM than static stretching (Sharman et al., 2006; Hindle et al., 2012). In PNF, a brief isometric contraction is performed, while the muscle is held stretched. Two major methods of PNF stretching are “the contract-relax” and “contract-relax antagonist contract” techniques (Youdas et al., 2010). The contract-relax method includes static stretching followed by contracting the target muscles isometrically, immediately followed by further stretching (Magnusson et al., 1996a; Kay et al., 2015, 2016). Greater ROM increase was reportedly obtained after contract-relax stretching than after static stretching (Magnusson et al., 1996a; Kay et al., 2015). The contract-relax antagonist contract method follows exactly the same procedure as the contract-relax method but requires an additional contraction of the antagonist (i.e., opposite muscle group being stretched) muscles during the stretch, before subsequent additional stretching of the target muscles (Youdas et al., 2010). Furthermore, it has been noted that some neuromuscular mechanisms are associated with significant improvements in ROM with PNF stretching (Funk et al., 2003; Guissard and Duchateau, 2006; Sharman et al., 2006; Hindle et al., 2012). For example, autogenic inhibition may occur during muscle contraction in PNF stretching (Sharman et al., 2006; Hindle et al., 2012). This neural pathway involves increased activity of Ib afferents from Golgi tendon organs inhibiting the spinal excitability of the stretched muscles (Sharman et al., 2006). In addition, reciprocal Ia inhibition may occur in the target muscles during PNF stretching when the antagonist muscle is contracted (Guissard and Duchateau, 2006). This is because the increased activity from Ia afferents of the antagonist muscle inhibits the spinal excitability of the target muscles via Ia inhibitory interneurons (Crone and Nielsen, 1989). However, the effects of modulation of these neural pathways on changes in ROM by static stretching remain unclear. Therefore, the identification of specific neural factors influencing changes in ROM by PNF methods would help modify static stretching techniques to improve acute and chronic responses.

Patterned electrical stimulation (PES) of the peripheral sensory nerve has been shown to induce plastic changes in the reciprocal Ia inhibitory circuit (Perez et al., 2003). More specifically, the strength of reciprocal Ia inhibition of Hoffmann-reflex (H-reflex) amplitude of the soleus (SOL) muscle was increased by PES applied to the common peroneal nerve (CPN), and it has been demonstrated that PES could modulate the activity of Ia inhibitory interneurons (Perez et al., 2003; Fujiwara et al., 2011; Kubota et al., 2015). Furthermore, uniform electrical stimulation (UES) to the CPN is ineffective for altering reciprocal Ia inhibition (Perez et al., 2003). The same number and intensity of pulses were set for UES as for PES, but the stimulation timing to the CPN differed between PES (10 pulses every 1.5 s) and UES (one pulse every 150 ms) (Perez et al., 2003). Considering that PES modulates reciprocal Ia inhibition of the SOL H-reflex, we combined PES and static stretching to test whether the reciprocal Ia inhibition would be a specific neural factor improving ROM acutely.

The purpose of the present study was to examine the effects of PES and static stretching on ROM and passive torque in plantarflexion muscles. We hypothesized that (1) combining PES and static stretching induces greater changes in ROM than UES and static stretching and (2) changes in ROM are correlated with changes in reciprocal Ia inhibition of the SOL H-reflex. To test these hypotheses, the effects of transcutaneous electrical nerve stimulation (i.e., PES and UES) on reciprocal Ia inhibition of the SOL H-reflex were examined in Experiment 1. The effects of transcutaneous electrical nerve stimulation and static stretching on ROM and passive torque were then examined in Experiment 2.

## 2. Materials and methods

### 2.1. Subjects

Fourteen healthy men (age, 20.8 ± 1.3 years; height, 170.8 ± 6.3 cm; body mass, 62.6 ± 6.8 kg) participated in the present study. They had no recent history of lower limb musculoskeletal injuries or neuromuscular disorders. The present study consisted of two experiments (Experiments 1 and 2), which were completed by all subjects. The procedure, purpose, risks, and benefits associated with the present study were explained to the subjects, and written informed consent was obtained from all of them. The ethics review committee for experimental research involving human subjects at Kyushu Sangyo University approved the experimental protocols (#2020-0005), which were conducted in accordance with the guidelines of the Declaration of Helsinki.

**Figure 1:**
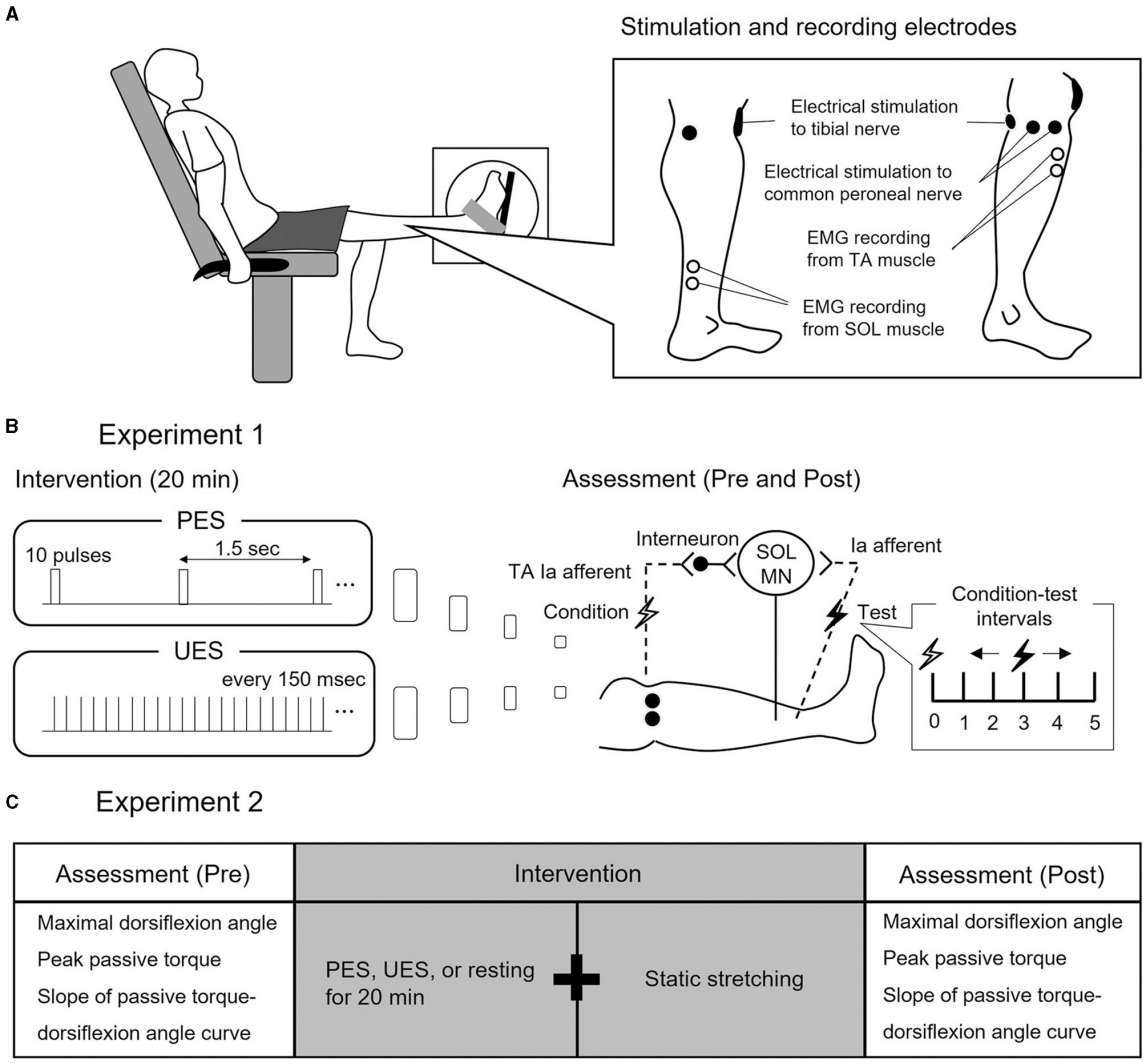
Experimental procedure and study design. **(A)** Experimental procedure. Subjects seated in a chair with knees fully extended. Electrode positions for electrical nerve stimulation and electromyographic (EMG) recording from soleus (SOL) and tibialis anterior (TA) are shown. **(B)** Study design of Experiment 1. Two interventions are used to stimulate different conditions of reciprocal Ia inhibition to SOL motor neurons (MN). Patterned electrical stimulation (PES) and uniform electrical stimulation (UES) are applied to the common peroneal nerve for 20 min. Assessment of reciprocal Ia inhibition is performed before and after PES or UES interventions. The SOL H-reflex is conditioned by stimulating the common peroneal nerve. Conditioning-test intervals indicate that the conditioning stimulus is applied before the test stimulus. **(C)** Study design of Experiment 2. The three interventions are static stretching after PES, UES, and prolonged resting without stimulation. Before and after the interventions, the passive ankle dorsiflexion test is performed to assess the maximal dorsiflexion angle, peak passive torque, and slope of passive torque.

The subjects were secured in an isokinetic machine (Humac Norm CN77, CSMI, Stoughton, USA) with their left knees fully extended (Figure 1A). The center of the lateral malleolus of the left foot was visually aligned to the rotational axis of the dynamometer. The left foot was firmly secured to the footplate of the dynamometer with a non-elastic strap. The angle of the back of the seat was 70° in relation to the floor. In the present study, the ankle angle was defined as 0° when the footplate was perpendicular to the floor.

### 2.2. Procedures

#### 2.2.1. Experiment 1

Subjects visited the laboratory on three occasions, with an interval between visits of at least 48 h. The first visit involved a familiarization trial, and the subsequent two visits assessed the effect of a 20-min intervention of transcutaneous sensory nerve stimulation on reciprocal Ia inhibition. The order of intervention was random for each subject.

Two interventions of stimulation paradigms were used to simulate different conditions of the reciprocal Ia inhibitory circuit, without producing dorsiflexion of the ankle joint (Perez et al., 2003) (Figure 1B): (1) PES was applied to the CPN with a train of 10 pulses at 100 Hz every 1.5 s and (2) UES was applied to the CPN with pulses that were uniformly spaced every 150 ms, set to the same number and intensity of pulses as in PES. In the two interventions of the stimulation paradigms, an electrical current was delivered using a constant current electrical stimulator (DS7R, Digitimer, Hertfordshire, UK) for 20 min (Fujiwara et al., 2011). The pulse duration was 1 ms, and the stimulation intensity was the motor threshold of the TA muscle (Perez et al., 2003).

**Figure 2:**
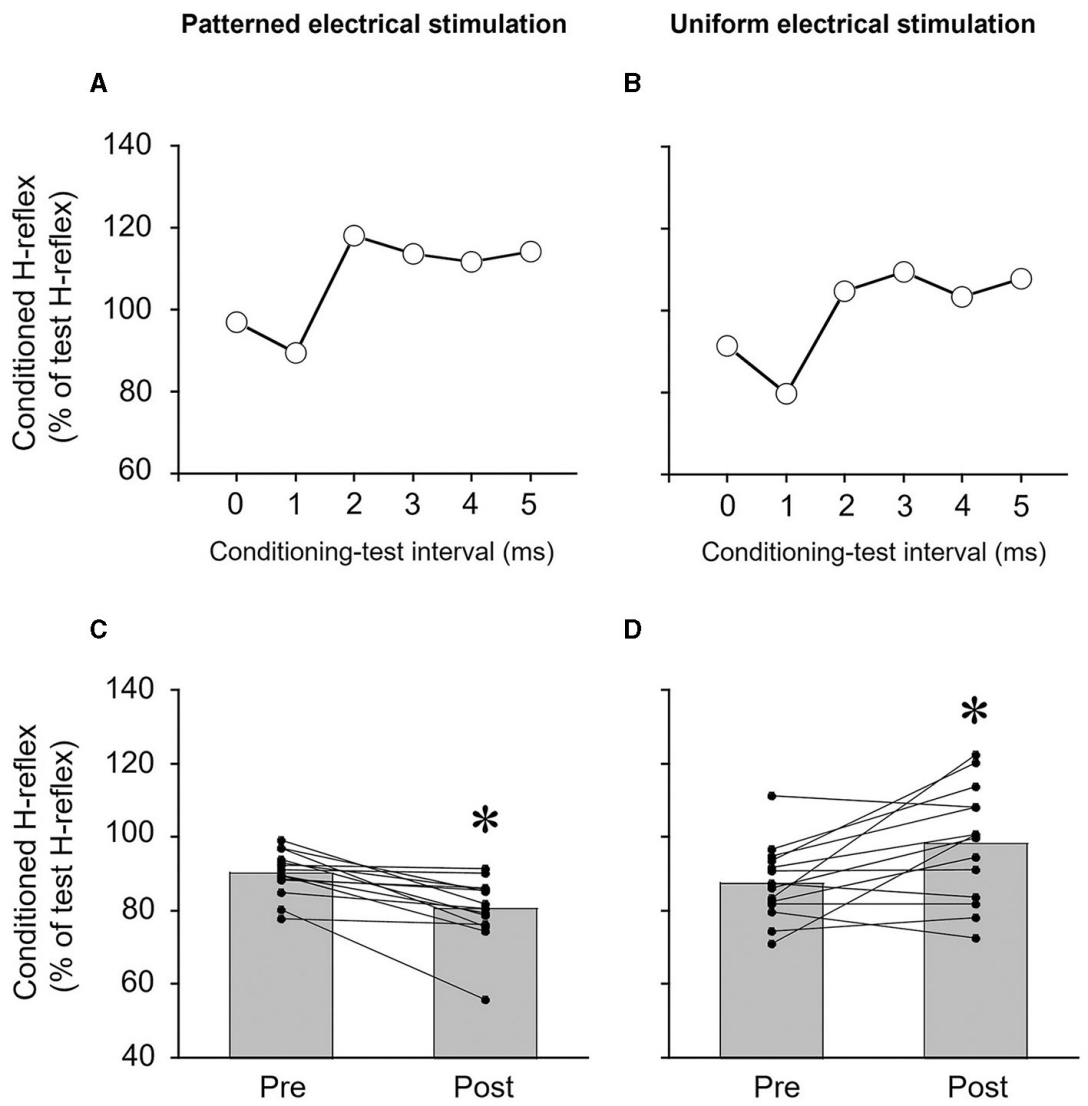
Typical examples of measurements of reciprocal Ia inhibition of the soleus H-reflex from a single subject **(A, B)** and the degree of reciprocal Ia inhibition of the soleus H-reflex **(C, D)**. **(A, B)** Time course of the effects of conditioning stimulation of the common peroneal nerve on the soleus H-reflex. The degree of reciprocal Ia inhibition was determined by conditioning stimulation of the common peroneal nerve to suppress the soleus H-reflex. In this subject, the conditioning-test interval for reciprocal Ia inhibition was set to 1 ms. **(C, D)** The effects of patterned electrical stimulation or uniform electrical stimulation on reciprocal Ia inhibition of the soleus H-reflex. Each black circle represents a data point in each subject. *p < 0.05 vs. pre.

Reciprocal Ia inhibition was measured before and after PES or UES (Figure 1B). It was assessed using the SOL H-reflex conditioning-test (C-T) paradigm (Figures 2A, B). Trials evoking the test SOL H-reflex were interleaved with trials in which a conditioning stimulus preceded the test SOL H-reflex. The SOL H-reflex was evoked by stimulating the posterior tibial nerve using a constant current stimulator (DS7R). The pulse duration was 1 ms. The cathode was placed at the popliteal fossa, and the anode was placed on the patella (Figure 1A). H-reflex and M-wave responses were measured as the peak-to-peak amplitude. Before each session, an H-reflex recruitment curve was obtained, and the H-reflex was expressed as a percentage of the maximal M-wave response (M_max_). To investigate the conditioning effect, the test H-reflex amplitude was adjusted to the same size (i.e., 20% of M_max_) (Crone et al., 1987). The conditioning stimulation to the CPN was stimulated with a rectangular pulse of 1-ms duration (DS7R) using a bipolar electrode that was placed distal to the head of the fibula (Figure 1A). The stimulation electrode was positioned carefully to avoid activating the peroneus muscles, thus ensuring more selective stimulation of the deep branch of the peroneal nerve. The stimulation intensity of the conditioning stimulus was set to the motor threshold of the TA muscle. The degree of reciprocal Ia inhibition was determined by conditioning stimulation of the CPN to induce short-latency suppression of the SOL H-reflex. The C-T interval for reciprocal Ia inhibition was varied from 0 to 5 ms in 1-ms steps, and then the optimal interval was determined and used before and after the intervention (1.5 ± 1.0 ms in PES; 1.5 ± 0.9 ms in UES) (Figures 2A, B). The SOL H-reflex was evoked every 10 s. Five conditioned and five unconditioned H-reflexes were averaged at each C-T interval. Conditioned and unconditioned H-reflexes were evoked in a random order.

#### 2.2.2. Experiment 2

Subjects visited the laboratory on three occasions separated by at least 48 h. The three visits included the following interventions: (1) static 3-min stretching after PES to the CPN, (2) static 3-min stretching after UES to the CPN, and (3) static 3-min stretching after prolonged resting without electrical stimulation (i.e., stretching without electrical stimulation intervention) (Figure 1C). The order of these interventions was random. PES and UES were applied to the CPN in the same manner as in Experiment 1.

**Figure 3:**
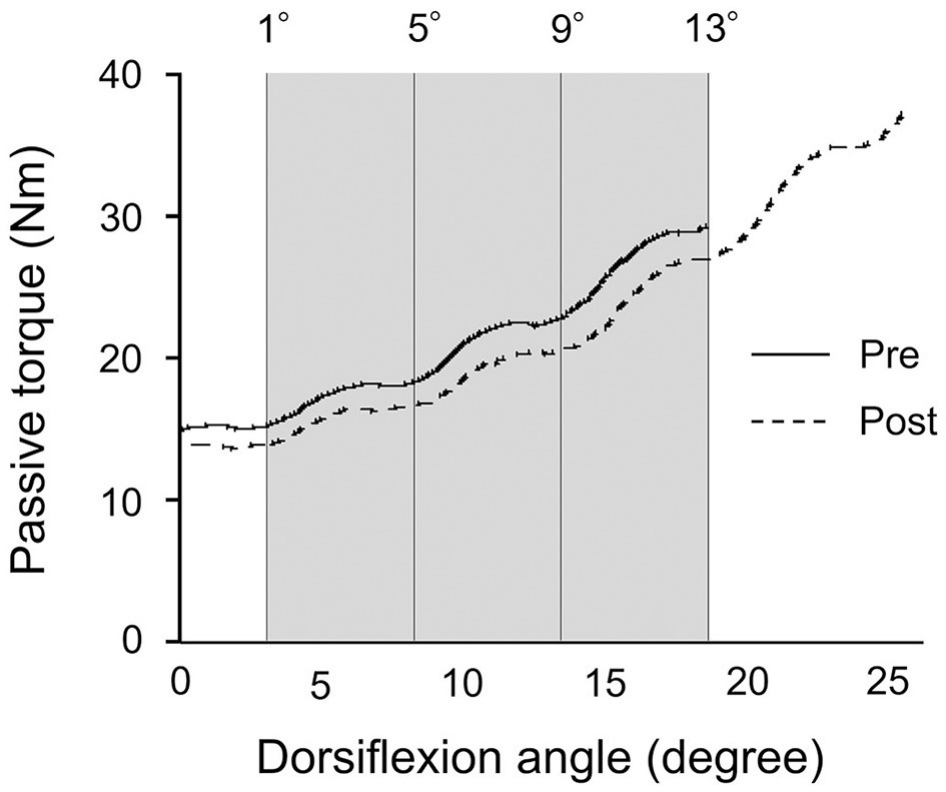
Typical example of the passive torque-dorsiflexion angle curve from a single subject during the passive ankle dorsiflexion test before and after patterned electrical stimulation and static stretching intervention. The gray area represents the calculation period for the final 13° that was common joint angles before and after intervention.

The passive ankle dorsiflexion test was performed before and after each intervention (Figure 1C). Passive dorsiflexion at 1°/s from 30° of plantarflexion to the maximal dorsiflexion angle determined based on the onset of discomfort for each subject was performed three times to define ankle ROM. The ankle joint angle was returned immediately to the plantarflexion position to avoid a stretching effect on stiffness of the muscle-tendon unit and stretch tolerance. Simultaneously, the passive torque on the footplate was recorded throughout the passive ankle dorsiflexion test. Subjects were asked to completely relax and not to exert any voluntary resistance. The maximal value of ankle ROM during the three passive ankle dorsiflexion tests was used for further analysis. The torque data were filtered with a zero-lag, 6-Hz Butterworth low-pass filter before passive torque was determined. Peak passive torque (i.e., stretch tolerance) was measured within a 250-ms epoch at the passive ankle dorsiflexion test (Kay et al., 2015). The slope of the passive torque-dorsiflexion angle curve (i.e., stiffness of the muscle-tendon unit) was measured at every fourth degree during the final 13° (i.e., at 1°, 5°, 9°, and 13°), which were common joint angles, before and after intervention (Figure 3) (Ryan et al., 2008).

Static stretching was performed using the dynamometer after transcutaneous electrical stimulation to the CPN or prolonged resting for 20 min. Since the PES effect on reciprocal inhibition lasts for at least 10 min (Perez et al., 2003), the static stretching protocol was done within 10 min. The static stretching involved three repetitions with no interval between repetitions. For each stretch trial, the left ankle was passively dorsiflexed from 30° of plantarflexion to the maximal dorsiflexion angle and fixed there for 60 s, and it was then returned to 30° of plantarflexion. The method for determining the maximal dorsiflexion angle was the same as for the passive ankle dorsiflexion test (i.e., onset of discomfort). The subjects were asked to relax their lower limb and to not exert any voluntary resistance.

### 2.3. Surface electromyographic recordings

Surface electromyographic (EMG) signals were recorded from the SOL, medial gastrocnemius (MG), lateral gastrocnemius (LG), and tibialis anterior (TA) in the left lower leg. Ag-AgCl electrodes (Vitrode F-150S, Nihon Kohden, Tokyo, Japan) with an inter-electrode distance of 20 mm were used for EMG acquisition from each muscle (Saito et al., 2019). The amplifier was set to a gain of 1,000-fold with a band-pass filter between 5 Hz and 1 kHz (AB-611J, Nihon Kohden). The EMG signals and torque signals were simultaneously sampled at 4 kHz using an AD converter (PowerLab, ADInstruments, Melbourne, Australia) and stored on a personal computer using software (LabChart 7, ADInstruments). The root-mean-square (RMS) values of EMG signals of the SOL, MG, LG, and TA during the passive ankle dorsiflexion test were determined for the initial 5° and for the final 5° of dorsiflexion, respectively (Mizuno, 2023). In each intervention, the RMS values of EMG signals in the lower leg muscles ranged from 21.6 to 41.6 μV for the initial 5° and 21.5 to 40.3 μV for the final 5° of dorsiflexion. Thus, it was ensured that subjects relaxed their lower legs during the passive ankle dorsiflexion test by surface EMG recordings.

### 2.4. Statistical analysis

The normality of the data distribution was investigated using the Shapiro–Wilk test. Since the distribution of the data was partly non-Gaussian, non-parametric statistical tests were used. In Experiment 1, the amplitude of the test H-reflex and the conditioned H-reflex of the SOL was compared before and after transcutaneous electrical nerve stimulation (i.e., PES and UES) using the Wilcoxon signed-rank test. In Experiment 2, differences in the ankle joint angle, peak passive torque, and the slope of passive torque were compared before and after intervention using the Wilcoxon signed-rank test. Changes in the maximal joint angle, peak passive torque, and the slope of passive torque were compared among three interventions by the Friedman test. Changes in passive torque at the fourth degree during the final 13° (i.e., at 1°, 5°, 9°, and 13°) were compared among three interventions using the Friedman test. When a significant effect was found, the Wilcoxon signed-rank test was performed. Spearman rank correlation coefficients (*r*_*s*_) were calculated to quantify the linear relationship between changes in the reciprocal inhibition of the SOL H-reflex (Experiment 1) and changes in the maximal joint angle, peak passive torque, and slope of passive torque by the PES and UES interventions (Experiment 2). The level of significance was set at a *p*-value of < 0.05, and the *p*-value for multiple comparisons was adjusted by the Bonferroni correction. The effect size (*r*), which was calculated by the *z-*value of the Wilcoxon signed-rank test and sample size, was categorized as trivial (0–0.09), small (0.1–0.29), medium (0.3–0.5), or large (>0.5).

## 3. Results

### 3.1. Reciprocal Ia inhibition

The size of the test H-reflex (% of M_max_) of the SOL was maintained at the same target amplitude between measurements of reciprocal Ia inhibition. The amplitudes of the test H-reflex before and after PES were 22.9 ± 6.5% and 22.2 ± 6.2% of M_max_, respectively (*p* = 0.594, *r* = 0.142). The amplitudes of the H-reflex before and after UES were 21.8 ± 4.6% and 22.2 ± 4.6% of M_max_, respectively (*p* = 0.875, *r* = 0.041).

Reciprocal Ia inhibition was significantly greater after PES (*p* = 0.001, *r* = 0.880) (Figure 2C) but significantly weaker after UES (*p* = 0.016, *r* = 0.645) (Figure 2D). The reciprocal Ia inhibition produced 9.7 ± 6.1% inhibition of the test SOL H-reflex before PES, and then it increased to 19.5 ± 8.8% after PES. The reciprocal Ia inhibition produced 12.4 ± 10.1% inhibition of the test SOL H-reflex before UES, and then it decreased to 1.7 ± 15.4% after the UES.

### 3.2. Maximal range of motion, stretch tolerance, and stiffness of the muscle-tendon unit

**Figure 4:**
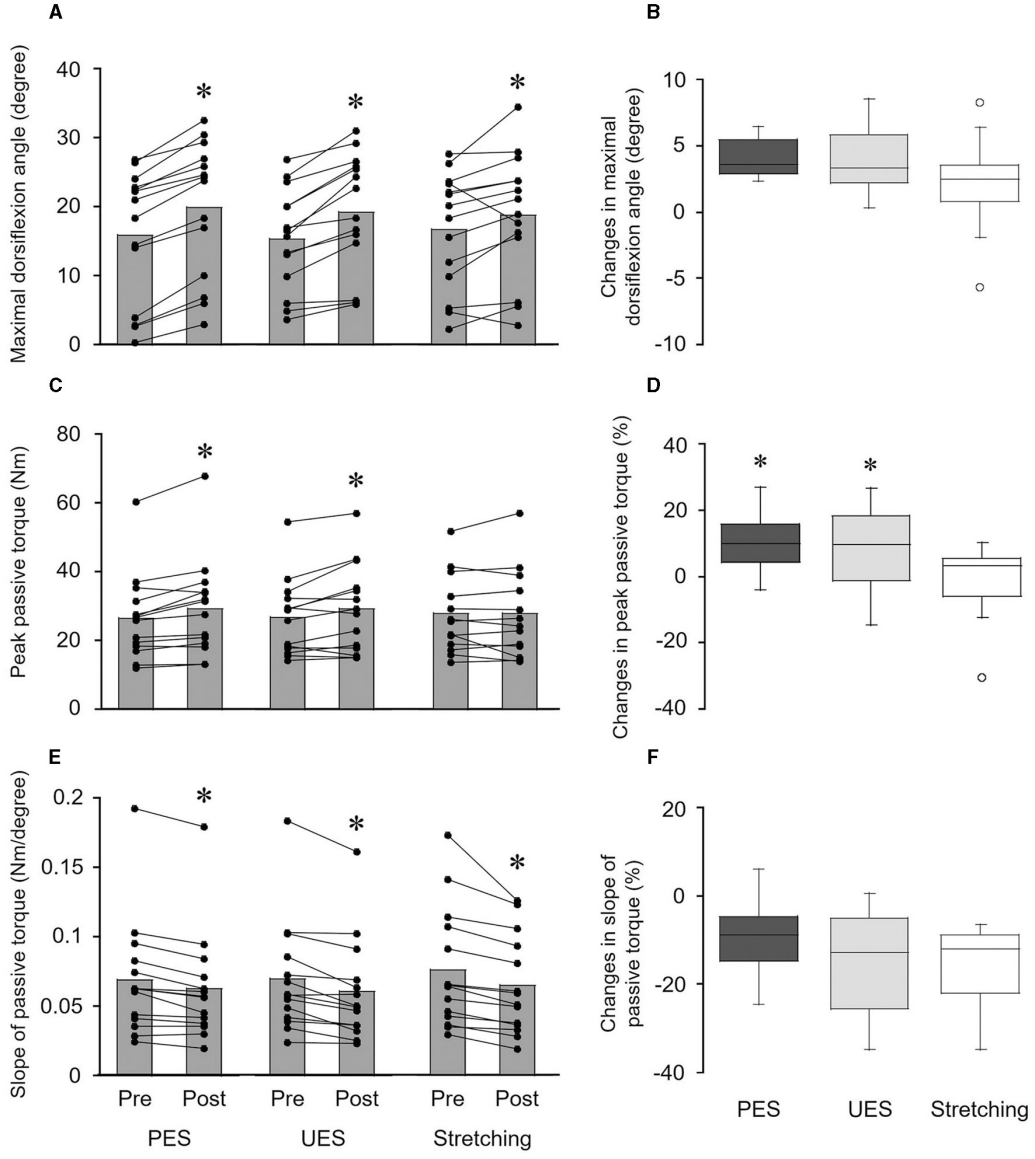
Effects of interventions on the maximal dorsiflexion angle **(A, B)**, peak passive torque **(C, D)**, and slope of passive torque **(E, F)**. The bar graphs show the maximal dorsiflexion angle **(A)**, peak passive torque **(C)**, and slope of passive torque **(E)** before and after interventions. Each black circle represents a data point in each subject. *p < 0.05 vs. pre. The box plots indicate the changes in the maximal dorsiflexion angle **(B)**, peak passive torque **(D)**, and slope of passive torque **(F)** by the interventions. The median values are shown with upper and lower quartiles, the whiskers represent the higher and lower extreme values, and the outliers are plotted as open circles. *p < 0.05 vs. stretching. PES, patterned electrical stimulation and static stretching; UES, uniform electrical stimulation and static stretching; stretching, static stretching without electrical stimulation.

A significant change in the maximal dorsiflexion angle was obtained before and after PES (*p* = 0.001, *r* = 0.880), UES (*p* = 0.001, *r* = 0.880), and stretching without electrical stimulation interventions (*p* = 0.026, *r* = 0.595) (Figure 4A). A significant difference in changes in the maximal dorsiflexion angle was not detected among PES (4.0 ± 1.4°), UES (3.8 ± 2.3°) and stretching without electrical stimulation (2.1 ± 3.3°) by the Friedman test (*p* = 0.071) (Figure 4B). No significant correlations were observed between the changes in reciprocal Ia inhibition and the maximal dorsiflexion angle in the PES (*r*_*s*_ = −0.121, *p* = 0.681) and UES (*r*_*s*_ = −0.262, *p* = 0.366) interventions.

Peak passive torque was significantly increased by PES (*p* = 0.004, *r* = 0.763) and UES (*p* = 0.030, *r* = 0.578) interventions but not the stretching without electrical stimulation intervention (*p* = 0.875, *r* = 0.041) (Figure 4C). A significant difference in the changes in peak passive torque was obtained among the three interventions by the Friedman test (*p* = 0.030) (Figure 4D). Changes in the peak passive torque were greater with PES (9.9 ± 8.2%; *p* = 0.027, *r* = 0.696) and UES (8.4 ± 12.0%; *p* = 0.033, *r* = 0.679) than with stretching without electrical stimulation (−0.7 ± 10.8%). No significant correlations were observed between the changes in reciprocal Ia inhibition and peak passive torque in the PES (*r*_*s*_ = −0.459, *p* = 0.098) and UES (*r*_*s*_ = −0.213, *p* = 0.464) interventions.

The slope of passive torque was significantly decreased by the PES (*p* = 0.002, *r* = 0.880), UES (*p* = 0.001, *r* = 0.864), and stretching without electrical stimulation interventions (*p* = 0.001, *r* = 0.880) (Figure 4E). A significant difference in changes in the slope of passive torque was not detected among PES (−9.3 ± 7.9%), UES (−14.0 ± 10.7%), and stretching without electrical stimulation (−15.2 ± 8.6%) by the Friedman test (*p* = 0.395) (Figure 4F). No significant differences in changes in passive torque were observed at the final 1° (*p* = 0.135), final 5° (*p* = 0.526), final 9° (*p* = 0.751), and final 13° (*p* = 0.607) by the Friedman test. No significant correlations were observed between the changes in reciprocal Ia inhibition and peak passive torque in the PES (*r*_*s*_ = −0.174, *p* = 0.553) and UES (*r*_*s*_ = −0.055, *p* = 0.852) interventions.

A significant difference was not observed in the dorsiflexion angles during static stretching among PES (15.9 ± 9.7°), UES (15.6 ± 7.9°), stretching without electrical stimulation (16.9 ± 9.5°) by the Friedman test (*p* = 0.878).

## 4. Discussion

The purpose of the present study was to examine the effects of PES and static stretching on ROM and passive torque in plantarflexion muscles. The results were that (1) an increase in ankle ROM and decreases in stiffness of the muscle-tendon unit in the PES intervention were similar to those with the UES and stretching without electrical stimulation interventions and (2) the changes in ankle ROM in the PES intervention did not correlate with the changes in reciprocal Ia inhibition of the SOL H-reflex by PES. These results did not support our hypotheses.

The results confirmed that PES enhanced the degree of reciprocal Ia inhibition (Figure 2C), and UES attenuated reciprocal Ia inhibition of the SOL H-reflex (Figure 2D). A previous study showed that reciprocal Ia inhibition was increased by 12–15% inhibition to 22% inhibition of the test SOL H-reflex amplitude by PES (Perez et al., 2003). It was demonstrated that changes in reciprocal Ia inhibition induced by PES could be attributed to changes in the excitability of Ia inhibitory interneurons (Kubota et al., 2015). Contrary to the effects of PES, reciprocal Ia inhibition of the SOL H-reflex was decreased 5% after UES (Perez et al., 2003). One of the possible mechanisms is that UES affects modification of supraspinal neural pathways. A previous study showed that inhibition of corticospinal excitability by transcranial direct current stimulation attenuated the strength of reciprocal inhibition and suggested decreased activity of cortical descending inputs to Ia inhibitory interneurons of the SOL (Fujiwara et al., 2011). Therefore, tonic activity of Ia afferents evoked by UES may modulate corticospinal descending inputs to Ia inhibitory interneurons.

Ankle ROM was increased after the PES intervention (Figure 4A), and an increase in ROM (4.0%) was similar to those with the UES (3.8%) and stretching without electrical stimulation (2.1%) interventions (Figure 4B). The changes in ankle ROM in the PES intervention did not correlate with the change in reciprocal Ia inhibition of the SOL H-reflex. These results suggest that enhancement of reciprocal Ia inhibition by PES is not effective for improving ROM by static stretching. Two potential factors may have caused these results. First, static stretching intensity during PES intervention (15.9°) was similar to that during the UES (15.6°) and stretching without electrical stimulation interventions (16.9°). It was demonstrated that stretching intensity determines the effectiveness of static stretching as the underlying mechanism that affects ROM (Freitas et al., 2015). Second, acute reduction of the stiffness of the muscle-tendon unit after PES intervention (−9.3%) was similar to that after the UES (−14.0%) and stretching without electrical stimulation interventions (−15.2%) (Figure 4F). Similar changes in the passive torque between interventions were also observed at the fourth degree during the final 13°. An ultrasonographic study showed that static stretching acutely reduces muscle stiffness, and the contract-relax technique of PNF stretching reduces both muscle stiffness and tendon stiffness (Kay et al., 2015). Unfortunately, the present study could not evaluate to which tissue the greater tension was applied after the PES intervention. Reciprocal Ia inhibition has been accepted as a neurophysiological explanation for acute ROM gains by PNF stretching (Sharman et al., 2006; Hindle et al., 2012). However, the results of the present study suggest that other neurophysiological mechanisms may be involved in the PNF technique, such as autogenic inhibition, passive mechanical properties of the muscle-tendon unit, and pain perception (e.g., gate control theory) (Sharman et al., 2006; Hindle et al., 2012).

The present study showed that the changes in stretch tolerance after the PES (9.9%) and UES (8.4%) interventions were greater than after the stretching without electrical stimulation intervention (−0.7%) (Figure 4D). The previous studies demonstrated that participants with more flexibility in joints had greater stretch tolerance than those with less flexibility in joints (Halbertsma and Göeken, 1994; Blazevich et al., 2012). It has been suggested that modification of afferent activity from the sensory receptors affects pain perception and changes in ROM (Magnusson et al., 1996b; Weppler and Magnusson, 2010). Interestingly, relatively similar changes in stretch tolerance were observed after contract-relax stretching compared to static stretching (Kay et al., 2015). These findings suggest that afferent inputs evoked by transcutaneous electrical nerve stimulation enhance the changes in stretch tolerance after static stretching. A previous study showed that electrical muscle stimulation over MG during static stretching improved ankle ROM but not stretch tolerance (Mizuno, 2023). This discrepancy may involve neural pathways following transcutaneous electrical stimulation between stimulation sites to muscles and nerves. Electrical stimulation over the nerve trunk can preferentially generate a sensory volley (large H-reflex and small M-wave), and electrical stimulation over the muscle belly can generate a motor volley (large M-wave and small H-reflex) (Bergquist et al., 2011). In addition, the strength of neural connections from afferent inputs to the sensorimotor cortex may also be involved. It has been suggested that the TA stretch reflex includes a larger long-latency component (i.e., transcortical pathway) than a short-latency component (i.e., spinal pathway) (Petersen et al., 1998). Assuming that the neural connection between Ia afferents of TA and the motor cortex is relatively strong, PES and UES to the CPN might effectively modulate stretch tolerance after static stretching compared to electrical stimulation to the tibial nerve.

It is well-known that movement-related cortical activity and afferent inputs, and their synchronization induce plastic changes in the central nervous system (Stefan et al., 2000; Wolters et al., 2003; Kaneko et al., 2022). A transcranial direct current stimulation study showed that cathodal stimulation improved the increase in ROM of the ankle joint but anodal and sham stimulation had no effect (Mizuno and Aramaki, 2017). Thus, PES and UES could modulate corticospinal excitability and pain perception, and they are possible mechanisms for improving the change in stretch tolerance. Furthermore, rhythmic burst stimulation to sensory nerves such as PES is an imitation of the sensory feedback from muscle spindles during locomotion (Perez et al., 2003). Specifically, the pulse train of 100 Hz was close to the firing rate of Ia afferents of dorsiflexor muscles in the early swing phase of locomotion (Geertsen et al., 2011), and the interval of 1.5 s is similar to the gait cycle cadence during slow walking in healthy individuals (Saito et al., 2017). The present study showed that improvement of stretch tolerance after static stretching had similar effects between the PES and UES interventions (Figure 4C). Therefore, modification of stretch tolerance induced by electrical stimulation of the sensory nerve might depend on the number of afferent inputs evoked by electrical nerve stimulation rather than the synchronization of cortical activity and afferent inputs.

There was a limitation to the measurement of reciprocal Ia inhibition in the present study. In the experiments, the reciprocal Ia inhibition of the H-reflex was measured from the SOL but not recorded from the MG and LG muscles. This was because the sensitivity of the H-reflex to facilitation and inhibition is related to the size of the test H-reflex (Crone et al., 1987). To address the conditioning effect on the H-reflex, the size of the test H-reflex was adjusted to 15–20% of M_max_ (Perez et al., 2003). In practice, the H-reflex amplitude of the SOL was large, but H-reflex amplitudes of MG and LG were relatively small. Therefore, the effect of changes in reciprocal Ia inhibition of the MG and LG H-reflexes by PES on ankle ROM and passive torque remains unknown. In addition, two separate experiments were designed because of the time limitation imposed by the effect of PES on reciprocal Ia inhibition. The effects of PES on reciprocal Ia inhibition last at least 10 min (Perez et al., 2003), and tests of reciprocal Ia inhibition and static stretching intervention could not be completed within 10 min in the same experiment. Therefore, the results in the present study involve the repeatability between two separated experiments to measure PES effects on reciprocal Ia inhibition.

In conclusion, transcutaneous electrical nerve stimulation and static stretching improve stretch tolerance regardless of the degree of reciprocal Ia inhibition of the SOL H-reflex. The present study investigated the combined effects of PES and static stretching on ROM and passive torque in the ankle joint. It was found that PES and static stretching improved ankle ROM, stretch tolerance, and stiffness of the muscle-tendon unit, with PES and UES having similar effects. A greater increase in stretch tolerance was observed after PES and UES compared to static stretching without electrical stimulation. These findings suggest that afferent inputs evoked by transcutaneous electrical nerve stimulation enhance the changes in stretch tolerance induced by static stretching.

## Data availability statement

The raw data supporting the conclusions of this article will be made available by the authors, without undue reservation.

## Ethics statement

The studies involving humans were approved by Experimental Research Involving Human Subjects at Kyushu Sangyo University approved the experimental protocols. The studies were conducted in accordance with the local legislation and institutional requirements. Written informed consent for participation in this study was provided by the participants' legal guardians/next of kin.

## Author contributions

AS contributed to the conceptualization, methodology, formal analysis, investigation, writing—original draft, and funding acquisition. TM contributed to methodology and writing—reviewing and editing of the manuscript. All authors contributed to the article and approved the submitted version.
